# The spiritual dimension of parenting a child with a life-limiting or life-threatening condition: A mixed-methods systematic review

**DOI:** 10.1177/02692163231186173

**Published:** 2023-07-17

**Authors:** Marijanne Engel, Marije A Brouwer, Nienke Jansen, Carlo Leget, Saskia CCM Teunissen, Marijke C Kars

**Affiliations:** 1 Julius Center for Health Sciences and Primary Care, Center of Expertise in Palliative Care Utrecht, University Medical Center Utrecht, Utrecht, The Netherlands; 2 Faculty of Medicine, Utrecht University, Utrecht, The Netherlands; 3 Department of Care Ethics, University of Humanistic Studies, Utrecht, The Netherlands

**Keywords:** Palliative care, pediatrics, parents, spirituality, systematic review

## Abstract

**Background:**

Spirituality refers to the dynamic dimension of human life that relates to the way that persons experience meaning, purpose, and transcendence. The complex task of parenting a child with a life-limiting condition may raise existential questions, which are easily overlooked by healthcare professionals.

**Aim:**

We explored how the spiritual dimension becomes manifest in parents of children in pediatric palliative care.

**Design:**

A mixed-methods systematic review was conducted, registered in Prospero (2021 CRD42021285318).

**Data sources:**

PubMed, CINAHL, Embase, PsycInfo, and Cochrane were searched for articles published between January 1, 2015 and January 1, 2023. We included original empirical studies that reported on spirituality of parents of seriously ill children, from parents’ perspectives.

**Results:**

Sixty-three studies were included: 22 North-American, 19 Asian, 13 European, 9 other. Studies varied in defining spirituality. We identified five different aspects of spirituality: religion, hope, parental identity, personal development, and feeling connected with others. All aspects could function as source of spirituality or cause of spiritual concern. Sources of spirituality helped parents to give meaning to their experiences and made them feel supported. However, parents also reported struggling with spiritual concerns. Several parents highlighted their need for professional support.

**Conclusions:**

Although studies vary in defining spirituality, reports on spirituality focus on how parents connect to their faith, others, and themselves as parents. Healthcare professionals can support parents by paying attention to the spiritual process parents are going through. More research is needed into how healthcare professionals can support parents of seriously ill children in this process.

**What is already known about the topic?**

- Spirituality is recognized as an essential component of high-quality palliative care.
- While spirituality in pediatric palliative has gradually gained some interest over the last few years, spiritual assessment and spiritual care do not seem to be a standard practice in care for families of children with life-limiting conditions.
- An in-depth understanding of the spiritual dimension of parenting a child with a life-limiting or life-threatening condition may result in spiritual care needs of parents being better recognized and met by healthcare professionals.

**What this paper adds?**

- With regard to spirituality, for parents caring for a child with a life-limiting or life-threatening condition, five aspects are important: how they connect to their religious beliefs; how they deal with hope; their parental identity; their personal development; and how connected they feel to others.
- All five aspects can function either as source of spirituality or cause of spiritual concern. However, there is a gap between the theory about spirituality, referring to spirituality as a process that is always there, and included studies referring to spirituality in terms of sources that give parents strength and causes of spiritual concern that bring confusion or frustration.
- Parents have needs for spiritual support, but their needs are often not explicitly stated.

**Implications for practice, theory or policy**

- All healthcare professionals should be sensitive to the spiritual dimension in caring for parents with children with life-limiting conditions and support these parents more adequately and systematically in their spiritual process.
- In future research, a clear definition of the concept of spirituality underlying a study would be helpful in bringing theory on spirituality and healthcare practice closer together.

## Introduction

Dealing with a life-limiting illness can present patients and their families with considerable challenges. The same is true for children, adolescents, young adults, and their families, when diagnosed with a life-limiting illness. The often unpredictable and sometimes erratic course of the disease, and the confrontation with how transient life is, give rise to spiritual questions and challenges.^1–4^ Spiritual well-being is very important to patients with an advanced illness.^1^ It can offer some protection against despair for adults at the end of their lives^5^ as well as for pediatric patients^6^ with an advanced illness, and the parents of ill children.^7^ Since the 1990s, a large and growing body of literature on palliative care in general has addressed the importance of spirituality as one of its four dimensions. Besides the physical, psychological and social dimension, the spritual dimension is also recognized as an indispensable component of high-quality palliative care.^8–11^

Although spiritual care is acknowledged as an essential component of palliative care,^12,13^ it remains a somewhat indistinct concept.^14–17^ Whereas spirituality used to refer predominantly to faith and religion,^15,18^ the concept has gradually become broader, signifying the universal process of searching for meaning in connection with the world around us.^12,14,19^ With the aim of reaching an international consensus, the EAPC Reference Group on Spiritual Care proposed an overarching definition of spirituality in palliative care that can be found under the heading ‘Definition of spirituality in palliative care’ in the methods section.^12^ We have chosen for this definition as guidance for this systematic review, because this broad definition suits well the current use of the concept of spirituality in palliative care and does not differ significantly from other important international consensus definitions.^16,20^The EAPC definition suggests that spirituality has not only religious and, or transcendental connotations, but also refers to the universal process of searching for meaning in everyday human experience.^19,21,22^

While spirituality in palliative care has gradually gained some interest over the last few years, the majority of the literature focuses on spiritual care for adult patients and their caregivers.^9,16^ Fewer studies have investigated the ways in which spirituality in daily life is perceived by parents of children in palliative care, although experiencing the life-limiting illness and death of one’s own child is known to be extremely challenging for parents.^3,4^ Spiritual assessment and spiritual care, meanwhile, do not seem to be a standard practice in care for families of children with life-limiting conditions.^23^ An in-depth understanding of the spiritual dimension of parents’ experiences of taking care of a child with a life-limiting or life-threatening condition is still lacking^24^ and may result in spiritual care needs remaining unmet.^25^

Therefore the main research question for this review was:

*How does the spiritual dimension, as defined by the EAPC Reference Group on Spiritual Care, becomes manifest in parents of children with life-limiting or life-threatening conditions?*

## Methods

### Design

We conducted a mixed-methods systematic literature review with a convergent integrated approach to the synthesis and integration of findings.^26^ This review is reported according to the updated Preferred Reporting Items for Systematic Reviews and Meta-Analysis (PRISMA) statement for reviews.^27^ The protocol was registered in Prospero (2021 CRD42021285318).

### Definition of spirituality in palliative care

Definition of spirituality adopted by the EAPC Reference Group on Spiritual Care

Spirituality is the dynamic dimension of human life that relates to the way persons (individual and community) experience, express and/or seek meaning, purpose, and transcendence and the way they connect to the moment, to self, to others, to nature, to the significant and/or the sacred.

The spiritual field is multidimensional, containing:

- Existential questions (concerning, e.g. identity, meaning, suffering and death, guilt and shame, reconciliation and forgiveness, freedom and responsibility, hope and despair, love and joy)
- Value based considerations and attitudes (i.e. the thing most important to each person, such as relationships to oneself, family, friends, work, nature, art and culture, ethics and morals, and life itself)
- Religious considerations and foundations (faith, beliefs and practices, the relationship with God or the ultimate).

The definition of the EAPC Reference Group on Spiritual Care was chosen as an overarching definition because it presents a broad view on spirituality.^12^

### Search strategy and databases

The search strategy was based on the “Palliative cAre Literature rEview iTeraTive mEthod” (PALETTE). This is an iterative method for the development of search strategies in palliative care literature for complex concepts such as spiritual care.^28^ An exploratory search was conducted in PubMed. The provisional search was adjusted several times following PALETTE methodology.^28^ The final search string was developed further in PubMed, optimized, and manually adjusted for use in other databases. On the 13th of October 2021, the original search was carried out in five online databases: PubMed, CINAHL, Embase, PsycINFO, and Cochrane (see Supplemental File 1 for search terms). The search was updated from October 2021 to January 1, 2023 on April 30, 2023.

### Inclusion and exclusion criteria

We included full-text English original empirical papers if they met the following criteria: they concerned pediatric palliative care; they concerned spirituality as experienced by parents; their findings fitted the EAPC definition of spirituality; they reported data from the parents’ perspective; and they were published between 1 January 2015 and 13th October 2021. The year 2015 was chosen as a starting point because the attention given to spirituality from the perspectives of children, their parents/families and healthcare professionals in pediatric palliative care, building on important international consensus definitions,^12,16,20^ is only been evident in recent years. The same is true of the development of guidelines.^29,30^

Duplicates were removed from the EndNote library using a method for de-duplication of database search results for systematic reviews in EndNote.^31^ The title and abstract screening and the follow-up screening based on the full articles were performed by two researchers (ME, NJ) and four researchers (ME, NJ, MB, MK) respectively. They used the screening capabilities within EndNote X9.^32^ Differences in the selection of articles which were potentially included were resolved in mutual consultation. When in doubt, an article was judged on its full text or in consultation with the research team.

### Data extraction and analysis

We developed a data extraction table that included the author, year, country, aim setting, design, sample, number of participants, data collection, and quality appraisal.

To analyze the findings from studies with qualitative, quantitative and mixed-methods research designs, we took a convergent integrated approach to the synthesis and integration of these findings.^26^ For the purpose of the synthesis, a conceptual overview of the results with regard to spirituality was made of each article and in this overview, statistical findings from included studies were transformed into descriptive summaries. This was performed by ME, MB, and NJ. An initial thematic structure was developed based on the overviews of the findings of articles published in 2020 and 2021 (found in the original search, *n* = 17) by ME and NJ with regular input from MB and MK for checking the themes found and their validation. Subsequently, all information from remaining articles, including articles from the updated search, was also analyzed thematically and placed along this preliminary structure. If necessary or desirable, themes were added or existing themes were merged. This resulted in a final structure of themes and subthemes reflecting the aspects of the spiritual dimension. During the later stages of analysis, the wider research team (also including CL and SCCMT) regularly validated the findings.

### Quality assessment

The assessment of the quality of the articles included was performed independently by two researchers (ME, NJ). Observational studies were assessed using an adapted version of the Cochrane risk of bias instrument, which was based on the Cochrane Bias Tool for intervention studies^33^ and was adapted for observational studies in cooperation with Cochrane Netherlands.^34,35^ For the quality assessment of qualitative articles, we used the CASP tool.^36^ Articles with a slightly lower quality assessment were also included given the exploratory nature of this systematic review.^37^

## Results

### Characteristics of the included studies

**Figure 1.**
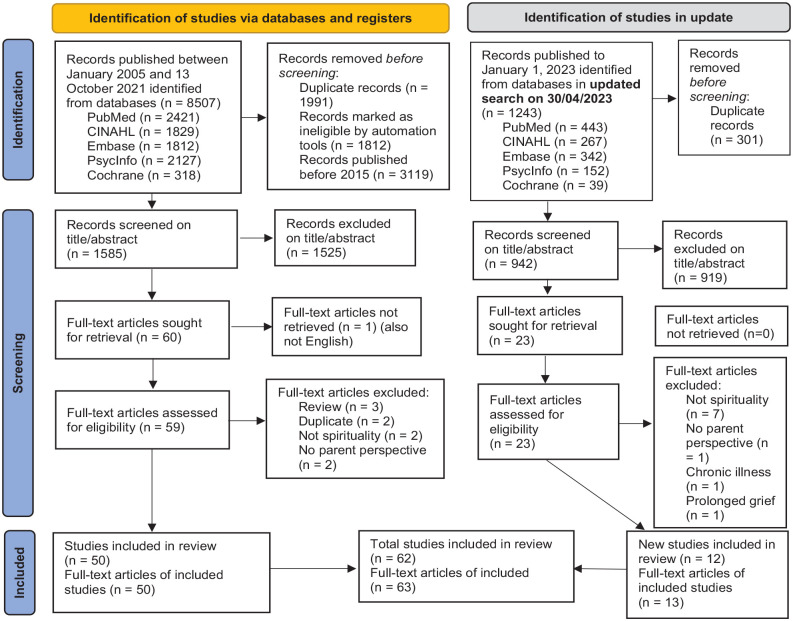
Flow diagram search strategy^27^ (2021-10-13 and updated search to 2023-01-01).

The final, updated search strategy resulted in 2527 unique articles covering the period from January 1, 2015, to January 1, 2023. Of these 63 were included meeting the criteria set out (see Figure 1).^38–100^

The vast majority of articles are based on qualitative research (*n* = 47).^38,39,41,42,44,46–52,54–59,61–63,65,66,68–70,72,74–78,80,82–84,86,88,90–97,99^ Eleven articles report a quantitative observational study.^40,43,45,53,67,71,73,85,87,89,98^ Five articles present a mixed-methods design.^60,64,79,81,100^ Most stud-ies (*n* = 22) were conducted in North America.^39,40,42–45,63,64,67,70–72,77–79,81,83,88,89,91,97,98^ Nineteen studies were from Asia.^38,41,46–49,58–60,69,73,74,76,82,86,87,94,96,99^ Thirteen were from Europe.^50,52,53,55,61,66,68,75,85,92,93,95,100^ Six studies were from South America,^54,57,62,80,84,90^, three from Australia.^51,56,65^ In total, the studies report on the perspectives of 2355 parents. An overview of the characteristics of the included studies is presented in Table 1.

**Table 1.** Characteristics of the included studies.

Author, year Country	Aim	Setting	Study design	Sample	Number of parent participants studied	Method of data collection	Quality appraisal
Qualitative studies							
Akaberian et al. (2021) IR	To discover the spiritual needs of mothers having children with cancer aged 1–12 years old.	Pediatric wards in specialized oncology hospital	Qualitative study: descriptive-exploratory design	Mothers having children aged 1–12 years old and the definite diagnosis of a type of cancer for at least 6 months. Nurses working in pediatric oncology wards	Mothers *n* = 12 Nurses* *n* = 3	Semi-structured in-depth interviews	9
Arora et al. (2021) US	To identify the priorities, existing supports, and opportunities for improvement in children’s end-of-life care, from parent perspective	Children’s hospital	Qualitative study: descriptive-exploratory design	Bereaved parents of children who had received care from the Comfort and Palliative Care Team, and whose children had died >18 months before study initiation	Parents *n* = 27 (mothers *n* = 17, fathers *n* = 10)	Semi-structured interviews in focus groups	9.5
Atashzadeh-Shoorideh et al. (2018) IR	To investigate the barriers and facilitators in providing spiritual care for parents who have children suffering from cancer.	State children’s hospitals	Qualitative study: descriptive-exploratory design	Parents of children suffering from cancer	Parents *n* = 15 (mothers *n* = 11, fathers *n* = 4)	Semi-structured interviews	8
Bally et al. (2021) CA	To explore family experiences and identify care and support needs across the illness trajectory of a child with a life-limiting or life-threatening disease	Mixture of settings	Qualitative study: interpretative description design	Parents who are the primary caregivers of a child with a life-limiting or life-threatening illness, aged between 2 weeks and 10 years. Health care and support providers from the publicly funded healthcare system, currently caring for children with life-limiting or life-threatening conditions	Parents: *n* = 7 (mothers *n* = 6, fathers, *n* = 1)Healthcare professionals* *n* = 9 Support providers* *n* = 2	Semi-structured interviews in focus groups	9.5
Bogetz et al. (2021) US	To examine the process by which parents adapt to their child’s serious illness and death	Children’s hospital	Cross-sectional qualitative study: descriptive-exploratory design	Parents, non-bereaved and bereaved, or legal guardians of AYAs between the ages of 14 and 25 years, who received treatment for advanced cancer ⩾18 months prior to recruitment	Parents *n* = 37 (mothers *n* = 29, fathers *n* = 8)	Demographic survey Semi-structured interviews in-person or by phone	8.5
Cai et al. (2020) CN	To understand nonreligious theistic parents’ spirituality and to explore how parents discuss death with their terminally ill children in mainland China.	Children’s hospital	Qualitative study: descriptive-exploratory design	1. Parents whose child was diagnosed with cancer and 2. parents whose child received palliative care and have bereaved for 3–12 months.	Parents *n* = 16 (mothers *n* = 11, fathers *n* = 5)	Semi-structured interviews	9
Cho-Hee et al. (2022) KR	To identify parental needs for pediatric palliative care and obtain their opinions on developing pediatric palliative care in South Korea.	University children’s hospital	Qualitative study: with deductive and inductive analysis approach	1. Parents actively caring for a child with life-limiting conditions (LTC) younger than 18 years, who were given the diagnosis within the last 10 years, and who had been admitted and treated by specialty care teams in the National University Children’s Hospital 2. Bereaved parents of children younger than 18 years who died from LTC longer than 3 months before inclusion.	Mothers *n* = 13 (actively caring for children *n* = 6, bereaved *n* = 7)	Semi-structured interviews	8.5
Chong L. et al. (2022) MY	To examine the clinical experiences of bereaved parents of children with a life-limiting illness to provide recommendations for quality care.	University hospital, and home palliative care service	Qualitative study: exploratory design	Parents of children with a life-limiting illness who had died >3 months before the interview	Parents *n* = 21 from 15 families (mothers *n* = 8 (from 8 families), fathers *n* = 1 (from 1 family), 6 mother/father couples (from 6 families))	Semi-structured in-depth interviews	9
Chong P.H. et al. (2021) SG	To explore how a good death for children can occur in the real-world context and identify factors influencing it.	Children’s hospital	Qualitative multiple-case study	A case refers to a child with life shortening illness in the last months of life, supported by caregivers (both professional and informal) within the health and social care system. The child had died between 6 and 24 months before the data collection.	Cases: *n* = 5 Including parents: *n* = 8 (mothers *n* = 5, fathers *n* = 3)Healthcare professionals* *n* = 14	In-depth, semi-structured interviews Before interviews, family caregivers were asked to bring photographs, videos or other physical items left behind as keepsakes. Their associated memories or meanings were explored at interviews.	9
Coad et al. (2015) GB	To explore perceived met and unmet needs of services and care of life-limited children, young people and families	Home	Qualitative study: Appreciative Inquiry (AI) design	Children and young people up to 25 years old with life-limiting illness and families including parents, legal guardians, family carers and siblings	Parents *n* = 59 (of whom mothers *n* = 44, fathers *n* = 8) Other key family members with a parent role (birth grandparents, foster grandparents and adoptive parents) (*n* = 7) Children* *n* = 18related to 51 families	Semi-structured, in-depth interviews with individuals	8
Collins et al. (2016) AU	To provide an in-depth exploration of the prevalent lived experiences of parents who are currently providing care for a child with a life-limiting condition in Australia	Pediatric hospice	Qualitative study: interpretative phenomenological design	Parents identified as the “primary caregiver” for one or more children/ adolescents (⩽18 years) diagnosed with a life-limiting condition and registered with a pediatric hospice	Parents *n* = 14 (mothers *n* = 12, fathers *n* = 2)	Semi-structured face-to-face interviews	10
Courtney et al. (2018) IE	To explore mothers’ perspectives of the experiences and impact on themselves and their family when their child has a life-limiting neurodevelopmental disability	Home	Qualitative study: subjective interpretative design	Mothers of children under 6 years of age with life-limiting neurodevelopmental disabilities, who receive support from a charitable service provider of home respite.	Mothers *n* = 12	Semi-structured interviews by telephone or at home	7.5
Dantas Jesuíno da Costa et al. (2018) BR	To understand the experiences of the mothers of children with cancer in palliative care.	Public hospital	Qualitative study: descriptive- exploratory design	Mothers of a child with cancer undergoing treatment at the public hospital	Mothers *n* = 20	Semi-structured interviews at the time of visits or when the child was sleeping	8.5
De Clercq et al. (2017) CH	To provide insight into how children, parents and physicians make sense of progressive childhood cancer, and to explore how this meaning-giving proces interacts with cultural dominant stories on cancer and dying.	Pediatric oncology centers	Qualitative study: dialogical narrative analysis	1. Children with progressive cancer with a poor prognosis, or for whom no curative treatment was available. 2. The parents of these children 3. their physicians	Parents *n* = 5 (mothers *n* = 4, fathers *n* = 1)Children* *n* = 6Physicians* *n* = 5	Open ended, face to face interviews	8
Donovan et al. (2022) AU	To understand the experience of families whose child had received specialist pediatric palliative care (PPC) in Australia, to ensure future service development and capacity building of health and social care professionals is informed by their lived experience.	Home	Qualitative study: descriptive-exploratory design using Discovery Interview methodology	Parents (1) whose child had been referred to the Children’s Health Queensland specialist Pediatric Palliative Care Service (PPCS) and was currently assessed as “stable,” or the bereaved parent of a referred child (more than 6 months bereaved) (2) whose child and family had participated in a pop-up education session. In this pop-up session parents, their child and family/community network are invited to share their experiences with local care professionals, in order to equip these professionals with skills and knowledge to address their needs.	Parents *n* = 11 (mothers *n* = 9, fathers, *n* = 2) (*n* = 5 palliative, *n* = 6 bereaved)	Telephone interviews guided by a “spine.” The interviewer invites the interviewee to discuss their experience of each area displayed in the spine.	10
Dos Santos et al. (2020) BR	To understand parental experiences of their relationships with providers at their child’s end of life with cancer and describe the manifestations of their grief.	Public hospital specialized in pediatric oncology and hematology	Qualitative study: philosophical hermeneutic approach	Bereaved parents whose child died at least 6 months prior to study participation Parents with their children, other family members, and with health care providers, in case a child was in hospital and two physicians confirmed that it would die within 6 months.	Families *n* = 24 Interviews bereaved parents *n* = 7 (mothers *n* = 6, fathers *n* = 1 )	• Interviews with bereaved parents • Observation of parents in their interactions with their children, other family members, and with health care providers.	9
					Observations Families* *n* = 18 (one overlap in family observation and interview)		
Doumit et al. (2019) LB	To understand the meaning of spirituality for parents of children with cancer in Lebanon without imposing any a priori categorization that may limit the field of inquiry.	Cancer center (inpatient and outpatient units)	Qualitative study: interpretive phenomenological approach	Mothers and fathers of children with cancer	Parents *n* = 11 (mothers *n* = 9, fathers *n* = 2)	In-depth and semi-structured interviews, participants were interviewed twice.	8.5
Effendy et al. (2022) ID	To explore the experiences of family caregivers of children living with cancer while receiving home-based pediatric palliative care	Home	Qualitative study: phenomenological design	Family caregivers of children living with end-stage cancer, no longer responding to curative treatment (as mentioned in the oncologist’s referral letter) while having received or receiving home-based pediatric palliative care at the moment of inclusion.	Parents *n* = 12 (mothers *n* = 11, fathers *n* = 1)	Semi-structured, in-depth interviews	8
Falkenburg et al. (2020) NL	To learn more about the specific features and function of the spirituality that is part of the confrontation with death	PICU of a children’s hospital	Qualitative study: narrative approach	Parents whose child, aged between 2 months old and 14 years, died in the pediatric intensive care unit (PICU), 5 years ago	Parents *n* = 36 (couples *n* = 16, mothers *n* = 3, fathers n = 1)	Unstructured face-to-face interviews with the opening question: “What happened to your child?”	9
Freitas et al. (2017) BR	To investigate the meaning attributed to spirituality and religiosity by mothers of hematologic cancer patients undergoing hematopoietic stem cell transplantation (HSCT).	hospital	Qualitative study: descriptive-exploratory design	Brazilian mothers in the role of informal caregivers of children diagnosed with leukemia or aplasia, and who were in the process of HSCT.	Mothers *n* = 10	Individual, phenomenological interviews	8
Hafez et al. (2021) SA (US)	To explore the perceptions of Muslim women primary caregivers, caring for a child at the end of life in Saudi Arabia.	Hospitals and home	Qualitative study: phenomenological design	Muslim Saudi Arabian women caring for a child under the age of 14, receiving end-of-life care, including mother, grandmother, aunt, sister, stepmother	Mothers or other women primary caregivers *n* = 24 (mothers *n* = 22, sister *n* = 1, nanny n = 1)	In-depth interviews	8.5
Higgs et al. (2016) AU	To explore parents’ perspectives of having a child with SMA type 1, from diagnosis to bereavement, in order to inform clinical practice by identifying aspects most meaningful to parents.	Home	Qualitative study:qualitative methods	Bereaved parents of children with SMA type 1	Parents *n* = 13 of 7 families (mothers *n* = 7, fathers n = 6)	In-depth interviews: 10 face-to-face, 1 by telephone)	9.5
Hurley et al. (2021) IE	To explore parental experiences surrounding the diagnosis of their child’s non-malignant life-limiting conditions	Mixture of settings	Qualitative study: descriptive design	Parents of children with non-malignant life-limiting conditions	Parents *n* = 23 (mothers *n* = 18, fathers *n* =5)	Semi-structured interviews	9
Jordan et al. (2015) IE	To explore individual experiences of transition, and the relevance of the concept of liminality to understand parents’ experiences of caring for a dying child.	Mixture of settings	Qualitative study: interpretive approach	Parents of children with a life limiting or life threatening condition who had died ⩾ 6 and ⩽ 24 months before the interview.	Parents *n* = 25 (mothers *n* = 16, fathers *n* = 9)	Face-to-face interviews	8.5
Jyothi Cornelio et al. (2016) IN	To explore the experiences of mothers on parenting children with leukemia	Hospital and home	Qualitative study: phenomenological design	Mothers of children between the age group of 1 and 16 years diagnosed with leukemia and undergoing chemotherapy in a Tertiary Care Hospital, either as inpatient or at the daycare center.	Parents *n* = 10 (not specified)	Semi-structured interviews The lead question was “What are your experiences having cared for a child who is undergoing treatment for leukemia in the hospital?”	7.5
Kamihara et al. (2015) US	To explore whether and how hope can coexist with a realistic understanding of a child’s poor prognosis, along with the nature of hopes held by parents with this knowledge.	Hospital	Qualitative study: qualitative methods	English-speaking parents of children <18 with advanced cancer that had recurred or was refractory to first-line therapy if they had a planned meeting with the child’s oncologist to discuss this diagnosis. Exclusion criterion: Parents of children with a first relapse of acute lymphoblastic leukemia, given higher possibility of cure.	Conversations between parents, children and physicians*: *n* = 36 Parent interviews: *n* = 32 (mother *n* = 26, fathers *n* = 6)	Audiotaping of conversations between parents, children and physicians about the child’s diagnosis of relapsed or refractory cancer. Semi-structured interviews with parents	7.5
Koch et al. (2022) US	To describe the challenges for parents serving as caregivers of children with Complex Life-Threatening Conditions (CLTCs) and their intersection with health care provider expectations through utilization and adaptation of the role theory framework.	University hospital and home	Qualitative study: descriptive design, secondary analysis of a longitudinal study on parent and provider decision-making for children with CLTC.	Parents of children (1) with three kinds of complex life-threatening conditions (CLTCs): extreme prematurity (<26 weeks gestation), complex congenital heart diseases, or genetic disorders requiring a hematopoietic stem cell transplant (BMT) (2) that were discharged from the hospital (3) were at home with their parents for any period of time.	Cases in this sub-study: *n* = 15 Each case included an infant, at least one parent, and at least three health care providers (physicians, nurses, nurse practitioners [NP], and social workers.* Number of parents in this sub-study: 15–30 (number of mothers and fathers unclear)	Repeated one-on-one semi-structured interviews during participation in the study (3–12 interviews per parent)	8
Lin et al. (2020) TW	To explore parental experiences in terms of spiritual practices when they face end-of-life decisions for adolescents with brain stem dysfunction on life support.	Pediatric intensive care unit at a medical center	Qualitative study: descriptive phenomenological design	Parents (1) whose child was on life support with brain stem dysfunction in a pediatric intensive care unit at a medical center and (2) who had participated in the child’s medical decision-making.	Parents *n* = 3 (a couple and 1 mother)	In-depth interviews	9.5
Lotz et al. (2017) DE	To investigate parents’ views and needs regarding pediatric advance care planning.	home	Qualitative study: descriptive design:	Parents (1) of children who had died from a severe illness (2) their child had died >6 months ago (3) who had readiness to talk about the child’s care and death.	Parents *n* = 11 (mothers *n* = 8, fathers *n* = 3, 2 couples)	Semi-structured face-to-face interviews	9.5
Lou et al. (2015) TW	To explore the essence of maternal experiences related to the anticipatory loss in families where a child is suffering from advanced-stage cancer.	Hospital and home	Retrospective qualitative study: phenomenological design	Mothers of a child who had died within the previous 3 years due to a brain tumor and were a member of the Childhood Brain Tumor Association-Taiwan (CBTA-Taiwan).	Mothers *n* = 10	In-depth interviews	8
Love et al. (2022) US	To characterize bereaved parents’ perspectives on the value of legacy activities; to describe parent recommendations for optimizing provision of legacy activities by child life specialists and music therapists.	Children’s research hospital	Qualitative study:qualitative methods	Parents (1) whose children were diagnosed with cancer, received treatment at the institution, and died 12 −24 months prior to the study; (2) who participated in a legacy-building activity from child life specialists and music therapists – focusing on the creation of lasting memories including activities such as hand or foot molds, memory books, songwriting, recordings, and scrapbooking. There is not a standardized procedure or policy dictating the timing or process for offering legacy interventions.	Parents *n* = 19 (mothers *n* = 17, fathers *n* = 2)	Semi-structured interviews during a follow-up telephone call	9.5
Malcolm et al. (2021) US	To understand how parents/legal guardians had utilized Religion and Spirituality (R/S) in the context prior to medical decision-making	Children’s hospital	Qualitative study: grounded theory approach	Parents who made significant medical decisions for their child, with a life-limiting or life-threatening decision.	Parents *n* = 24 (mothers *n* = 19, fathers *n* = 5)	Semi-structured interviews	10
Misko et al. (2015) BR	To understand the family’s experience of the child and/or teenager in palliative care and building a representative theoretical model of the process experienced by the family.	Hospital and home	Qualitative study: Symbolic Interactionism and Theory Based on Data approach	Families of children and/or teenagers that are in monitoring or have been accompanied by the Pain and Palliative Care team, the child and/or teenager has a “limiting condition of life” or a “life threatening” condition, documented in their chart and the family has been informed about this, which also should be documented in the clinical record. Families whose son had died and had been accompanied by the Pain and Palliative Care team.	Parents *n* = 20 (mothers *n* = 15, fathers *n* = 3, 1 grandfather and 1 paternal aunt) (from 15 families)	Semi-structured interviews	7.5
Nafratilova et al. (2018) ID	To explore the parents’ experience in caring for children with cancer under palliative care condition	Home	Qualitative study: descriptive phenomenological design	Parents (1) who had experience in caring for children with cancer under palliative care conditions where the main therapy had stopped (2) who were the primary care givers for their children	Parents *n* = 10 (mothers *n* = 8, fathers *n* = 2)	In-depth interviews with open-ended questions	5.5
Nicholas et al. (2017) CA	To explore parental spirituality in the context of pediatric cancer with a poor prognosis	Hematology /oncology unit of a pediatric hospital	Qualitative study: longitudinal grounded theory design	Parents of a child who was being treated in the hematology/oncolo-gy unit, with a poor prognosis	Parents Time 1: *n* = 35 (mothers *n* = 26, fathers *n* = 9) Time 2: *n* = 30 (mothers and fathers not specified) Time 3: *n* = 27 (mothers and fathers not specified)	Semi-structured, individual interviews. Participants were interviewed three times.	9
Palacios-Espinosa et al. (2021) CO	To explore the meanings of the experience of being a caregiver in Colombia for boys and young men with Duchenne Muscular Dystrophy (DMD)	Home	Qualitative study: phenomenological approach	Caregivers of boys and young men (between 9 and 17 years, one 21 years old) with DMD living in Bogotá, Colombia.	Mothers *n* = 7	Unstructured interviews	8.5
Pishkuhi et al. (2018) IR	To understand the parents’ experience in caring for a child with cancer and thereby help the health team and health decision-makers to understand their biological and mental status.	Hospital; and home	Qualitative study: phenomenological approach	Parents who have (1) a cancer child under the age of 15 years; (2) in the family, there should be only one child with cancer and the other family members should not have cancer; (3) the sick child should not have a known chronic disease other than cancer	Parents: *n* = 13 (not specified)	In-depth interviews	8
Schaefer et al. (2021) US	To explore meaning-making pre-bereavement among children with advanced cancer and their parents	Hospital and home	Qualitative study: longitudinal approach	Parents and their child, (1) who had advanced cancer (2) was aged between 5 and 25 years	Parents: *n* = 37 (mothers *n* = 26, fathers *n* = 11) Children* *n* = 24(33 families)	Semi-structured interviews at the baseline of a larger longitudinal study	8
Silva et al. (2019) BR	To describe the phenomenon of parents or other family caregivers caring for children without current therapeutic treatment to cancer and its repercussion for home care.	State public hospital and home	Qualitative study: descriptive approach using the Social Representation Theory	Parents or other family caregivers who care for children without current treatment to cancer, in the palliative phase.	Parents or other family caregivers *n* = 12 (mothers or other woman caregivers (not specified) *n* = 11, grandmother, *n* = 1)	Semi-structured interview script containing 3 open questions of individual application.	7.5
Smith et al. (2018) CA	To increase the understanding of parental caregivers’ needs from multiple perspectives to keep their hope possible and to develop an intervention that can be implemented by healthcare professionals	Mixture of settings	Qualitative study: Delphi approach using an exploratory method of interpretive description	Persons who had expertise in caring for children who have a life-threatening or life-limiting condition including parental caregivers (birth, adoptive and foster parents), that were (1) English speaking, (2) ⩾18 years, and (3) the primary current or bereaved caregiver for their infant or child between the ages of 3 months to 15 years at the time of illness.	Parents *n* = 21 (not specified)	A Delphi study consisting of three rounds of survey questions Round 1: Participants were asked to brainstorm ideas and make suggestions for strategies or activities for each of the four subprocesses of the Keeping Hope Possible theory of Bally et al., 2014 Round 2: Consisted of summarizing and reviewing the rankings of themes from Round 1. Round 3: Participants were asked if they agreed with the results of round 2. The methodology of Interpretive Description was used to identify final themes.	8.5
Somanadhan and Larkin (2016) IE	(1)To understand and interpret parents’ experience of living and caring for a child with MPS. (2) To examine the knowledge and understanding of MPS from the perspective of parents. (3)To explore the impact of regular hospitalization of children living with Mucopolysaccharidosis (MPS) on family life.	Home	Qualitative study: hermeneutic phenomenological approach	Parent of a child, adolescent or young adult (0–24 years old), with a diagnosed MPS condition (I-IX); Parents of children who attend the National Center for Inherited Metabolic Disorders (NCIMD); Parents currently residing in the Republic of Ireland and receiving treatment for their child in Ireland; Those able to communicate through English	Parents: *n* = 8 (a total of 19 interviews) (mothers *n* = 7, fathers *n* = 1)	Three time point serial in-depth interviews over a 17-month period	9
Szabat (2020) PL	To analyze the experience of hope that appears in a parents’ blog presenting everyday life while caring for a child with Trisomy 18	Home	Qualitative case study	Parent of a 10-year old son with Trisomy 18	Mother *n* = 1	In-depth investigation of a blog that was posted regularly over a period of around 10 years and described life with her son. 215 (out of 664) posts were chosen for analysis of hope issues.	8
Taib et al. (2021) MY	To explore the challenges faced by parents with children who have complex neurological conditions, their coping strategies, needs, and expectations.	Specialized pediatric hospital	Qualitative study: descriptive- exploratory design	Parents of children with life-limiting neurological illnesses, who have been in the wards for longer than 2 weeks at the moment of inclusion in the study	Parents *n* = 11 (fathers *n* = 7, mothers *n* = 4)	Semi-structured, in-depth interviews	9
Verberne et al. (2019) NL	To provide insight into the most prominent experiences of parents caring for a child receiving palliative care at home and to identify the main coping strategies they adopt to allow themselves to continue with their daily lives.	Home	Qualitative study: Interpretive design	Parents who (1) were Dutch-speaking (2) were aged ⩾18 years (3) had a child with a life-limiting or life-threatening condition, primarily residing at home (4) were referred to the Emma Children’s University Hospital pediatric palliative care team (PPCT)	Parents of 24 children *n* = 42 (mothers *n* = 24, fathers *n* = 18) Interviews: *n* = 47 11 parents were interviewed after their child’s death, 5 out of these 11 interviews were second interviews).	Semi-structured in-depth interviews (single or repeated)	10
Wang et al. (2019) TW	To describe the lived experiences of parents caring for a child with cancer at the end of life (EOL) in Taiwan with the goal of obtaining insights into the ability to introduce palliative care sooner in future cases.	Hospital	Qualitative study: descriptive phenomenological design	Parents who (1) had lost their child (age <20 years) to cancer more than 6 months earlier, (2) had cared for their child during the EOL period, (3) had no diagnosis of mental illness, (4) were able to communicate in Taiwanese and Mandarin and (5) were willing to participate in this research study and provide written consent	Parent *n* = 10 (mothers *n* = 6, fathers, *n* = 4)	Private interviews with open ended questions	9
Weaver et al. (2021) US	To provide a framework for conceptualizing how palliative care teams can communicate with parents in ways that support their personal identity as a “good parent.”	Mixture of settings	Qualitative case study Using the Good Parent Beliefs Construct and the Communication Theory of Identity (CTI)	Parent of a 16-year old son with metastatic Ewing’s Sarcoma	Father *n* = 1	In-depth investigation of a case	4.5
Yang et al. (2016) TW	To explore the lived experiences of anticipatory loss of parents of children with Spinal muscular atrophy (SMA) and the commonality of their multiple representations.	Home	Qualitative study: phenomenological design	Parents of school-age children with type I or II SMA.	Parents *n* = 19 (mothers *n* = 10, fathers *n* = 9) related to 10 children	In-depth interviews	8.5
Quantitative Studies							
Arutyunyan et al. (2018) US	To understand whether parents of children admitted to pediatric intensive care unit (PICU) would want their physicians to ask about their religious or spiritual beliefs. The secondary objective included exploring whether the child’s underlying illness or parental characteristics influence the responses to our primary objective.	PICU at a tertiary care university-affiliated medical center	Quantitative study: Observational cross-sectional design	Parents whose children had been admitted for more than 48 h to PICU at a tertiary care university-affiliated medical center.	Parents *n* = 162 (mothers *n* = 131, fathers *n* = 24, guardians *n* = 5, role unknown *n* = 2)	Survey	3
Bedoya et al. (2021) US	To explore potential demographic and medical factors associated with parent perceptions to better understand current care practices before, during and after their child’s death from cancer, and identify cohorts in need of additional support.	Mixture of settings	Quantitative study: Observational cross-sectional design	Bereaved parents whose child had died from cancer and were members of a closed social media (Facebook) group (“Parents who lost children to Cancer”).	Parents *n* = 178 (mothers *n* = 132, fathers *n* = 42, grand parent n = 2, role unknown *n* = 2)	The 46-item End-of-Life Practice Preference Survey designed to collect retrospective information, consisting of closed questions about availability and utilization of types of support and one open question: “*What, if any, other supportive services do you think should be offered to families? Please note when you feel these services should be provided.”*	1
Boyden et al. (2021) US	To explore how parents rate and prioritize domains of pediatric palliative and hospice care in the home setting (PPHC@Home) as the first phase of a larger study that developed a parent-reported measure of experiences with PPHC@Home.	Home	Quantitative study: cross-sectional assessment design Discrete choice experiment (DCE): used in healthcare to elicit preferences from participants (patients, payers, commissioners) without directly asking them to state their preferred options.	Parents whose children were currently receiving PPHC@Home, as well as bereaved parents whose children had previously received PPHC@Home.	Parents *n* = 47 (mothers *n* = 44, fathers *n* = 3)	Discrete choice experiment (DCE), with maximum difference (MaxDiff) scaling to obtain a quantitative estimate of the relative importance of each domain (i.e., domain importance scores), as rated by parents, and to rank the PPHC@Home domains by order of the parent rated importance score	3
Czyżowska et al. (2021) PL	To explore if mothers struggling with a child’s cancer experience posttraumatic growth and whether there is a relationship between spirituality and posttraumatic growth	University Hospital and Child Health Centre	Quantitative study	Mothers of children with cancer in the phase of treatment who had been staying with their child in the hospital for at least 2 weeks.	Mothers *n* = 55	• Posttraumatic Growth Inventory • Self-description Questionnaire of Spirituality • A short questionnaire on demographic variables and information on the child and his/her disease	3
Janvier et al. (2016) CA	To examine parental goals and hopes after a diagnosis of trisomy 13 or 18. Factors associated with survival among families who experienced a live birth were also investigated.	Mixture of settings	Quantitative study	Parents of children who live(d) with trisomy 13 or 18 who were part of online (English language) parent support groups in different countries (US, Canada, UK, other countries)..	Parents *n* = 261 (of 216 children) (fathers *n* = 202, mothers *n* = 59)	Self-reported questionnaire	2
Kelly et al. (2016) US	To evaluate patient caregivers’ perceptions of the extent to which their religious and spiritual (R/S) needs were assessed and addressed in the hospital.	Pediatric academic hospital	Quantitative study: cross-sectional design	Primary caregivers of pediatric inpatients referred for palliative care <1 year prior for a life-limiting illness. Patients were <25 years old and were diagnosed >4 weeks before the interview.	Parents *n* = 25 (mothers *n* = 19, fathers, *n* = 6)	Survey that measured the importance of religion and spirituality to the subject and the extent of R/S support by the subject’s religious community and medical team, including the nine-item Attitudes Toward God Scale-9.	2
Latha et al. (2016) IN	To identify and describe the symptoms (medical/social/ emotional) that most concerned parents during the last days of their child’s life and to identify the strategies parents found to be helpful during this period.	Pediatric hemato oncology unit in a hospital.	Quantitative study	Parents who lost their child aged between 0 and 18 years of cancer treated in our institution and whose child had died ⩾3 months before the study period.	Parents *n* = 10 (number of mothers and fathers unknown)	Interviews with a validated prepared questionnaire	2
Picci et al. (2015) IT	To compare the emotional burden and coping strategies of parents of children with rare diseases with those of parents of children with chronic diseases.	Mixture of settings	Quantitative study	1) Parents of children with rare diseases (CRD) and 2) Parents of children with chronic diseases (CCD) and 3) communication of the diagnosis ⩾1 year prior to enrollment	Parents of CRD *n* = 55 (mothers *n* = 30, fathers *n* = 25) Parents of CCD *n* = 56 (mothers *n* = 30, fathers *n* = 26)	A self-report study using, • the Satisfaction with life Scale (SWLS), a scale to measure quality of life, subjective well-being and satisfaction with life • The Profile of Mood States (POMS) that assesses mood states by evaluating six affective-emotional components of well-being • The Coping Orientation to Problem Experienced (COPE) to assess coping strategies • The Hamilton Rating Scale for Depression (HAM-D) • The Hamilton Rating Scale for Anxiety (HRSA)	4
Rao et al. (2022) IN	To ascertain the need for a pediatric caregiver support group based on a survey that explored the unmet needs of caregivers of children with cancer.	Hospital	Quantitative study: cross-sectional design	Primary caregivers of pediatric cancer patients aged 3–15 years, undergoing treatment, who were on follow-up with the pediatric oncologist between July and August 2019.	Parents *n* = 17 (mothers *n* = 9, fathers *n* = 7, uncle *n* = 1)	A 33-item survey assessing needs in seven domains: physical concerns, emotional concerns, family-related concerns, social concerns, logistics-related concerns, informational concerns and spiritual/religious concerns	1
Siden and Steele (2015) US + CAN	To explore the impact of a child’s neurological or rare genetic life-threatening condition on the affected child and his/her parents.	Mixture of settings	Quantitative study: cross-sectional observational approach The article describes early findings of the study.	Families of children (0–19 years old) with progressive neurological of chromosomally based condition with impairment of the CNS.	Parents *n* = 390 (mothers *n* = 249, fathers *n* = 141) Children* *n* = 275Siblings* *n* = 70(from 258 families)	Cross-sectional baseline results from an observational longitudinal study, with surveys (monthly assessments). • Monthly parental assessment of child’s symptoms • Pediatric Evaluation of Disability Inventory • Semi-annual survey about parental health and wellbeing (family functioning, marital satisfaction, health status, anxiety, depression, stress, burden, grief, spirituality and growth)	4
Wiener et al. (2020) US	To assess the degree to which parents felt prepared to address their child’s medical problems and emotional needs at the EoL, as well as the implications from lack of preparation.	Mixture of settings (where the child had died) in the US, Canada, Australia, the Philippines, and European countries	Quantitative study: Retrospective cross-sectional approach	Parents who (1) lost a child to cancer (2) whose child was 24 years old or younger at death (3) were member of a closed social media group (“Parents who lost children to cancer”)	Parents *n* = 131 (mothers *n* = 98, fathers *n* = 29, grandparent *n* = 2, other *n* = 2)	Retrospective survey with also open-ended questions.	3
Studies with mixed-methods design							
Eskola et al. (2017) CH	To understand parents’ experiences and needs during a child’s end-of-life care at home and to identify systemic factors that influence its provision.	Home	Mixed methods study: concurrent embedded design	Parents of children who had died due to a cardiologic, neurologic, oncologic illness or died within the first 4 weeks of life, who received end-of-life care at home, had spent ⩾21 days at home in the last 4 weeks of life and had died a minimum of 1 year ago.	Survey: Parents *n* = 66 Interviews: Parents *n* = 10 (mothers *n* = 7, fathers *n* = 1, couples n = 2)	• Survey about parental experiences and needs • Semi-structured interviews	Quantitative part: 2 Qualitative part: 9
Haley et al. (2016) US	(1) To identify the strengths of primary parent caregivers of children receiving in-home hospice/palliative care (2) to assess if the Haley Transcultural Strengths Assessment Interview Guide increases parent’s realization of the importance and utilization of their strengths.	Home	Mixed methods: Qualitative study: Grounded theory design Quantitative study: cross-sectional design	Parents caring for their child at home supported by home based care	Parents *n* = 8 (7 mothers and 1 grandmother)	• Interviews: following completion of the Pre-Personal Strength Rating Scale, and guided by the Haley Trans-cultural Strength Assessment Interview Guide for Parent Caregivers • Surveys: Pre- and Post-Personal Strengths Rating Scale, a Likerttype scale quantifying the individual’s perception of his or her strengths.	Quantitative part: 2 Qualitative part: 5.5
Michelson et al. (2022) US	To analyze parents’ views of their interactions with Social Workers (SWs) and chaplains during their child’s pediatric intensive care unit (PICU) admission and how parents perceive SW and chaplain involvement in decision making for their critically ill child with cancer.	University children hospital	Multi-methods study: Surveys Qualitative study	Parents with children (1) that had had either a cancer diagnosis or had undergone a hematopoietic cell transplant (HCT) (2) if the child had been in the pediatric intensive care unit (PICU) <7 days (3) that had one or more of the following characteristics: Pediatric Index of Mortality 2 (PIM2) score ⩾4%, expected PICU admission of >3 days (based on attending PICU physician input), previous PICU admission, plan to consult palliative care, or involvement of palliative care.	Quantitative Parents of 18 children *n* = 24 (mothers *n* = 17, fathers *n* = 7) Qualitative Parents *n* = 6 (number of mothers/fathers unknown)	Quantitative data Surveys 1: • Background characteristics • Religiosity/spirituality, assessed using “yes/no” questions • Social support, assessed using the - Information Support-Short Form 8a (Support SF 8a) PROMIS measure Survey 2 given within 3 days of PICU discharge: • asked parents if they spoke with an SW and/or chaplain during the PICU admission, and if so, whether the SW and/or chaplain helped them. • if they would speak with an SW and/or chaplain again if a future PICU admission occurred. Survey 2 further asked parents: • (1) identify “the most important decision made for their child in the PICU,” • (2) complete the Decision Regret Scale (DRS) • (3) indicate who on the healthcare team helped them talk with their doctors about that decision • (4) complete 10 items from the Pediatric Family Satisfaction in the Intensive Care Unit (pFS-ICU) Survey Qualitative data Semi-structured interviews at PICU discharge	Quantitative part: 3 Qualitative part: 7.5
Mooney-Doyle et al. (2018) US	(1) To describe parents’ expectations for themselves in terms of prioritizing tasks and goals for children with life-threatening illnesses (LTIs) and healthy siblings; (2) to compare parents’ tasks and goals for children with LTIs and healthy siblings; (3) to describe parenting in terms of the process of prioritizing tasks and goals for children with LTIs and healthy siblings	Children’s hospital	Mixed-method study: Concurrent cross-sectional design	Parents whose children with life-threatening illnesses lived with them at least part-time (2–3 d/week), were admitted to an inpatient unit for treatment of the LTI or associated complications, and who had other children who lived with them at least part-time.	Parents who participated in the interviews *n* = 31 (mothers *n* = 25, fathers *n* = 6) Parents (from the interview group) who provided complete quantitative data *n* = 29	Semi-structured interviews (Aim 1 and 3) Discrete choice experiment (Aim 2)	Quantitative part: 3 Qualitative part: 9
Zimmermann et al. (2022) CH	To gain a deeper understanding of what is important to parents in order to guide the further evaluation and improvement of PPC and end-of-life (EOL) care services. Mainly aimed (1) to report on qualitative data from the nationwide Paediatric End-of-LIfe CAre Needs in Switzerland (PELICAN) study (2012–2015) that were concerned with parental experiences about the care at their child’s EOL, and (2) to explain published quantitative questionnaire data by combining them with unpublished qualitative interview data from the PELICAN study.	Hospitals Long-term institutions Pediatric community care services	Mixed methods study: explanatory sequential approach.	Parents who showed maximal positive or extremely negative results in the Parental PELICAN questionnaire (PaPEQu), representing a statistical outlier in the negative range, i.e. third quartile + 1.5*interquartile range in the PaPEQu survey, and who had already consented at the time of recruitment to possibly participate in an interview, were contacted by phone. The four main diagnostic categories of the PELICAN study (cardiology, neonatology, neurology, and oncology) were additionally used as strata to ensure the same representation as in the survey sample.	Quantitative Final PELICAN sample Parents *n* = 200 (112 mothers and 88 fathers from 135 families). Qualitative (this study) Parents of 20 children *n* = 30 (mothers *n* = 18, fathers *n* = 12)	Quantitative data • The Parental PELICAN questionnaire (PaPEQu) was used in the PELICAN study (2012–2015) It described and explored parental perspectives of their child’s EOL care and the perspectives of the healthcare professionals involved (results have been previously published). • In this study quantitative data of the 30 interview participants are reported. Qualitative data • Semi-structured interviews (unpublished qualitative data from the PELICAN study)	Quantitative part: 3 Qualitative part: 10

* For this study, we focused on the perspectives of parents.

## Quality appraisal

For qualitative studies (*n* = 47), and for qualitative components of mixed-methods studies (*n* = 5), the median quality score of the report was 8.5 out of 10 (range 4.5–10). For observational studies (*n* = 11), and the quantitative parts of mixed-methods studies (*n* = 5), the median quality score was 3.0 out of 7 (range 1–4). A detailed presentation of the quality assessments by the researchers is presented in Supplemental File 2, Tables A.2.1 to A.2.4.

## The spiritual dimension of parents’ experiences in pediatric palliative care

### Description of spirituality

There was a wide variation in the definitions of spirituality. In 49 articles spirituality was not specified. In the other 14 articles a wide variation in the definition or description of spirituality was found. In 10 articles two or more different aspects of the EAPC definition were given. In four articles spirituality was only described as religion. An overview of the characteristics of the descriptions of spirituality is presented in Supplemental File 3.

### Aspects and how they manifest themselves in parents

We identified, through thematic analysis, five aspects that are important to parents with regard to spirituality. These were: how they connect to their religious beliefs; how they deal with hope; their parental identity; their personal development; and how connected they feel to others. Sometimes parents mentioned these aspects as source of spirituality. It strengthened them in various ways, for example by helping them to find meaning in their difficult situation, to retain some hope and control, function as a cause of spiritual concern bringing confusion, inner conflicts, and, or frustration to parents. This could lead to feelings of senselessness, despair, and loneliness. Thematic analysis also revealed that parents often did not explicitly express their need for spiritual support or maybe researchers reported little about these needs. A summary of findings is presented in Table 2.

**Table 2.** Summary of findings, from the parents’ perspective.

Aspects	How they manifest in parents		
	Sources of spirituality	Causes of spiritual concern	Spiritual support needs
Religion	Finding meaning	Confusion	Parents have diverse needs related to religion
Hope	Feeling strengthened	Loss of hope leading to distress and despair	Sustaining hope
Parental identity	Endeavoring to be a good parent	Struggling with inner conflicts	Feeling acknowledged in the struggles that they face. Feeling supported in parental participation
Personal development	Transformation and post-traumatic growth	Existential challenges in personal life due to loss	Care that fits the child’s and family’s situation. Confidence in the care team
Connection with others	Bringing comfort and strength	Loneliness	Feeling encouraged in connecting to others. Feeling protected

### Religion

#### Religion as a source of spirituality

A large number of studies reported on religion as a source of spirituality. Parents mentioned ways in which their belief in God, or the ultimate helped them to deal with existential challenges and find meaning in their difficult situation. It helped them to make sense of the incomprehensible^69,74,88^and to believe that whatever happens, God knows what is best for them^62^ or it offered comfort through the meaning of being part of a larger purpose.^83,88^ Religious belief could also help parents give meaning to their experiences and made them feel supported when coping with the reality of their child’s illness.^41,44,46,61–63,69,74,76,78,82–86,88,89,93–96,99^ Parents found meaning in their religion because they felt a sense of connection with God or the ultimate,^38,41,44,46,61–63,69,74,76,78,82,83,85,86,88,89,93,95,96,99^ felt suppor-ted,^38,41,46,62,76,78,82,83,85,89,96,99^ experienced guidance,^69,74,88^ or hope.^44,46,62,63,69,74,82,83,86,93^ The studies also included a variety of religions.^38,40,41,45,49,58,61–63,71,73,74,78,83,94,96,99^ In addition some parents with no previous religious belief reported still finding solace in religion and prayer in times of difficulty.^46^

Religion could also help reconcile parents to their inability to protect their child from the serious prognosis. It helped them to accept their lack of control,^83^ and to transform their grief over the prospect of losing their child to living life to the fullest with their child.^99^

Finally, religion helped parents to give meaning to the death of their child.^46,62,93^ The belief in an afterlife or being reunited after death brought comfort.^63,78,93,95^ This was true even if the parents did not believe in an afterlife.^61^ Some parents also believed that the illness of their child was a test of their faith. This helped them to remain strong.^63,88^

#### A cause of spiritual concern – confusion

Religion could also bring confusion, inner conflicts and, or frustration to parents.^41,44,46,73,74,78,83^ Some parents reported feeling a sense of divine, or spiritual, abandonment.^48,83^ They felt punished through the illness^78^ and blamed God or the ultimate for the illness of their child.^78^ Others reported inner conflicts between the information that they had been given by professionals and what they believed was the will of their God or the ultimate.^74^ Uncertainty about the afterlife could cause great confusion and grief to parents.^46^ Other parents wondered if they were being punished by their God or through the disease of their child.^44,73,74^ The psychological stress caused, for example, by not understanding why God created this problem for them, sometimes threatened parents’ connection with their religion^41,46,78^ or prophets or saints.^38^ Some parents lost their faith as a result.^41^ Finally, some parents reported that spiritual practices were too demanding during this time.^83^

#### The need for spiritual support related to religion

Relatively few articles reported on the need for support with regard to religious beliefs despite being a major theme in the spiritual experience of parents.^38,40,41,43,58,74,78,79,87,94^ A North-American survey study among parents whose children had been admitted for more than 48 h to PICU at a tertiary academic medical center^40^ showed that parents’ (*n* = 162, 82% Christian, 10% religious without category, 6% nonreligious and 2% other) wishes were mixed about whether their child’s physician should ask about their spiritual or religious beliefs. Parents views were divided equally between agreeing, disagreeing, and being unsure. A few recent studies reported on the needs of parents for facilities for spiritual practices including contact with a professional. Some parents indicated that during hospital admission of their child they need a prayer room,^87^ the availability of a spiritual/religious person,^43,79,87^ or more time for praying.^94^

### Hope

#### Hope as a source of spirituality

Hope, derived from seeing the good, that is having good days or living longer than expected, strengthened parents.^75^ It helped them to focus on the care of their child, and thus, to provide them the best quality of life.^80^ Hope also helped parents maintain a combative spirit,^55^ perceiving the illness of their child, against all knowledge, as temporary.^55,61,75,91^ Kamihara et al.^70^ found that parents were able to balance hope with realistic and more distressing expectations regarding the prognosis and progression of the illness.

#### A cause of spiritual concern – loss of hope

Losing hope was a common feeling for some parents.^44,91,93,99^ It could lead to strong feelings of senselessness, anguish, sadness, fear, anger, and despair.^54,65,80,88^ Many parents indicated that accepting the child’s illness, while at the same time staying hopeful, could also be challenging.^44,54,65,80,88,91,93,99^ Some parents reported losing all feelings of hope after the diagnosis. They questioned why this was happening to their loved one.^44,54^

#### The need for spiritual support related to hope

Several parents highlighted their need for professionals to strengthen them by sustaining hope,^94^ focusing on positive thoughts,^75,91^ taking things day by day, hoping for the best each day, and demonstrating positivity.^91^

### Parental identity

#### Endeavoring to be a good parent as a source of spirituality

Endeavoring to be a good parent was another source of spirituality that might strengthen parents and help them to find meaning in their experience. It presented parents with a destiny that is to care for their child.^63,76,78,84,93,95,97,99^ The endeavor to be a good parent manifested itself in several ways. Parents went to great lengths to provide the best care for their child. If cure was no longer an option, then they endeavored to grant their child a good and dignified death.^73,75,76^ Being acknowledged in this role, for example by being involved in decision-making processes, helped parents cope and gave them a sense of control.^75,100^

Being a good parent was also manifested in their efforts to strive for what they considered a good death for their child. Being able to let their child go helped parents to achieve this.^49^ Taking care of their child’s legacy was another way for parents to find meaning.^77,88^ Parents also indicated that farewell-activities drew them, their child and others involved, closer together and helped them experience a good death.^49,75,81,96,99^

#### A cause of spiritual concern – struggling with inner conflicts

Most parents preferred to fully embrace their parental role by caring for the child themselves,^95^ and when they experienced difficulties in doing so^52,68^ it caused feelings of distress and despair.^52,54,69,72,86,88,90,93,96,98,99^ With regard to treatment, parents reported inner conflicts between doing “too little” and doing “too much”,^49,67,75,95^ and frustration in case their expertise was questioned during hospital admission of the child^72^ or their child was not addressed respectfully.^100^ Lacking control made parents feel unprepared, scared,^46^ and vulnerable.^68^ Caring for their child may have reduced emotional and spiritual distress but parents often experienced it as a difficult and exhausting task.^95^

Finally, parents reported inner conflicts related to what they considered important for their child. Providing care at home meant they had to navigate a lack of family privacy and control.^49,50^ Parents felt torn between wanting to stay physically close to their child and their other commitments, such as jobs or the interests of siblings and peers.^49,50,72,97^

#### The need for spiritual support related to parental identity

Parents expressed a wish for professionals to support them in their parental role. This could be achieved by acknowledging the struggles they face,^44^ by emphasizing that they can still do a lot for their children and be good parents,^75^ by providing meaningful information, acting as an advocate for their participation,^39,50^ and by sensitively introducing the possibility of legacy-building.^43,77^ Parents indicated that this helped them to feel more in control, to make the best decisions for their child,^66,75,91^ and not to feel disconnected from their child.^91^

### Personal development

#### Personal development as a source of spirituality

Several articles mentioned personal development as a source of spirituality.^49,52,53,55,62,68,85,89^ The illness was initially seen as a period of fear, chaos, and loneliness.^55^ However, this phase could sometimes function as a welcomed liminal haven.^55^ Parents mentioned elements of “restitution” in other words restoring things to their normal life, considering the illness, against better judgment, as temporary,^55^ and an opportunity for personal growth.^62,72^ Several parents even experienced positive life changes as a result of post-traumatic growth.^53,84,89^ For some parents, their experiences led to a fundamental shift in their personal values, their approach to life, and their relationships with others^62,68^ or themselves.^62^ Other parents coped with illness by framing the situation in a more positive way.^85^ Being able to let their child go for some parents signified a turning point in the disease trajectory where they moved from doing everything possible to doing everything “right” in order to ultimately reduce suffering.^49^ Parents also described achieving some meaning to their situation by giving something back, for example by doing voluntary work.^52^

#### A cause of spiritual concern – existential challenges in personal life

Parents experienced an increasingly pronounced dislocation between two worlds from the moment they received their child’s diagnosis. There was the new world which revolved around the caring and other requirements of their child and their old world that did not.^56,66,68,100^ This brought existential challenges in their personal life. Parents put personal needs second, experiencing limited opportunities to continue their own life because of their child’s needs.^95^ They had to constantly adapt to fulfill their parental duties.^54,55,89,95^ This meant they experienced losses in several ways^51,52,56,80^ such as the loss of normality.^52,99^ They also described feeling torn, guilty, and heartbroken, because they might not have lived up to the expectations they had set for themselves and their families.^56,68,76,81,92,95^

#### The need for spiritual support in relation to existential challenges

Several articles reported a need for structures that provided stability when facing existential challenges.^39,49,50,66,78,85,95^ Parents, for example, do not want to be left alone by the care team after receiving their child’s diagnosis.^66^ They need confidence that the care team, whether specialized in palliative care^39,44,80^ or not,^50,85^ takes responsibility for supporting them in what they consider important.^39,44,49,50,66,78,80,85^ Parents, therefore, need adequate care that fits their child and family situation and they actively guide healthcare professionals to provide such care.^49,95^

### Being connected to others

#### Being connected to others as a source of spirituality

Feeling connected to others was mentioned as a great source of spirituality. Parents were strengthened, especially, through the bond with their child.^61,100^ The connection that parents felt with their child, even in the absence of verbal communication,^61^ helped parents to know that their child was still fighting,^61^ or when the child decided “it was time to go”.^61,95^ After the death of their child, ordinary events could have a transcendent meaning for parents, signifying the ongoing feeling of connection with their child.^61^ For example, through seeing a symbolic representation in birds or butterflies, or music from toys that spontaneously started playing without prompting it.^61^

Support from family, friends, their faith community, and other parents with similar experiences, also brought parents comfort and strength.^41,44,48,58,61,72,76,80,83,86,94^ Parents spoke of stronger relationships, and a greater appreciation of people in their network, to the detriment of material values.^62^

Parents’ connection with healthcare professionals enabled them to feel that the medical team was with them.^76,85,100^ This helped parents to feel tranquil and supported.^57–59,76^ Adequate communication by healthcare professionals was not simply a matter of professional responsibility, but was integral to the parents’ sense of themselves as effective in a world in which they had often lost previous competencies.^68^

#### A cause of spiritual concern – loneliness

Despite the meaningful connections parents described, many parents also described feeling disconnected or lonely from the world around them. They felt that people around them did not understand the complexities of their lives.^51,52,54,56,76,80,92^ Parents missed activities that would allow them to connect with other families.^51,52^ In the period after the loss of their child, they struggled to redefine themselves in relation to the outside world.^60^ Parents also described a certain distance from their child, because they felt their expertise was not sufficiently recognized by the healthcare professionals and their role as a caregiver was unclear.^91^ Some parents reported that their marital relationships had become markedly strained^72,92,94^ because of a lack of social time together, or because they did not share the goals they had for their child.^52,54,76,81,86^

The suffering and loneliness experienced by parents challenged permanent bonds with the healthcare professionals as parents often changed their previous evaluations of these relationships.^55^ Parents could feel abandoned by the professionals after their child died as professional support stopped and there was no further regular contact.^57,60,92^

#### The need for spiritual support in relation to connections with others

Many parents expressed the need to be encouraged to connect with others.^42–44,46,52,68,72,80,90^ Supportive relationships provided security and protective factors for parents’ bereavement.^57,68^ Connecting to other parents who went through similar struggles could be helpful for parents.^44,47,81,94^ Being connected to others also involved parents’ relationships with the care team. Parents expressed a need for a supportive, compassionate relationship with the care team, whether or not they were specialized in palliative care.^39,47,56,66,94,98^ They wanted to feel “held” and protected by care providers, even when these relationships were not clarified through verbal communication. One mother described how she felt nurses’ non-physical presence. The knowledge that they were around, even though the nurses respected her privacy by leaving her alone at times, helped her to feel secure.^57^

## Discussion

### Main findings

This systematic review explored how the spiritual dimension, in articles that included findings that fit to the EAPC definition, becomes manifest in parents of children with life-limiting or life-threatening conditions. We found five aspects that are important to parents with regard to spirituality: religion, hope, parental identity, personal development, and the connection with others. All can both function as a source of spirituality and as cause of spiritual concern. Sources of spirituality, such as hope and endeavoring to be a good parent, help give parents strength and meaning to their experiences. The causes of spiritual concerns, such as loneliness and feeling disconnected, bring confusion, inner conflicts and, or frustration to parents. Several parents highlighted their need for professional spiritual support, for example, by helping them maintain hope or by encouraging them to connect with others.

### What this study adds

A wide variation in the description of spirituality was found in the articles. These ranged from no description at all, to several definitions of the concept of spirituality. In all the descriptions of spirituality given, we identified one or more dimensions that are also found in the EAPC definition: existential questions, value based considerations and/or religious considerations and foundations. In several articles, one or more dimensions of spirituality were found in the description of the coping strategies of parents or in the psychological, emotional, or social aspects of care. This conceptual ambiguity has already been addressed both in several studies on adult care^12,14,15^ and in a few studies on parents’ experiences in pediatric palliative care.^4,101,102^ The broad view on spirituality contained in the overarching definition used by the EAPC Reference Group on Spiritual Care touches on many important aspects and refers to a continuous spiritual process in persons.^12^ However, in our review, parents’ language in included studies often refers to specific elements of meaning-making that would give them strength or be a cause of spiritual concern as if spirituality is not a process that is always there. Haufe et al.,^5^ for example, mention religion as a source of spirituality that leads to the process of meaning-making. Spirituality in itself is not only positive, but also a process of struggle and often found in everyday things.^5,19^ We found, obviously driven by the research topics of the studies in question and the underlying views on the concept “spirituality”, that parents talk about the spiritual process in terms of sources that give them strength and causes of spiritual concern that bring confusion or frustration. Parents’ use of language can lead healthcare professionals to think causally about spirituality rather than about spirituality as a process. In future research, a clear definition of the concept of spirituality underlying a study would be helpful in bringing theory on spirituality and healthcare practice closer together.

We also found that parents’ needs for spiritual support were often not explicitly stated. It is, therefore, not surprising that healthcare professionals do not recognize these needs and, thus, may not respond to them. Our findings show that further elaboration of the way in which parents experience spirituality, in both less or more visible forms, and the way healthcare professionals can recognize and respond to the process parents are going through, will be helpful in being sensitive to the spiritual dimension and supporting parents more adequately and systematically in their spiritual process.

We have the impression that most parents were aware that, together with their child and relatives, they had to walk this road themselves. In the spiritual dimension, they asked, mainly, that healthcare professionals care, and show they care, about their child and family. This finding is in line with a literature review of parents’ experiences of palliative care in which it was found that, in their communication with healthcare professionals, parents wish to be respected as experts in the care of their child but also have the support they need recognized.^4^ The need for good communication between parents and healthcare professionals is true for all four dimensions of pediatric palliative care, but certainly for the spiritual dimension. We hope the findings of our research will contribute to a deeper understanding of parents’ needs for spiritual support and the ways in which healthcare professionals can better recognize these needs and communicate properly with parents about them.

### Strength and limitations of the study

A strength of our study is that we included articles from five continents finding commonalities and cultural differences between spiritual beliefs and practices. However, caution is advised regarding generalizability of our findings to other parts of the world than the Netherlands and Europe because of differences in views on spirituality. Some other limitations of this systematic review should be taken into account. The terminology used in this systematic review refers to the EAPC definition and thus to a broad approach to spirituality, but was not labeled as such in part of the included studies. In addition, the concept of spirituality in a young discipline like pediatric palliative care is often not sharply defined.^28^ As a result, there is a gray area in the selection. We are not sure how to interpret this, because our selection approximates practice, where spirituality often is found in everyday life and everyday language.^5^ However, it could also serve as a disadvantage to leave the inclusion of findings to the interpretation of the research team, even when was sought for agreement among the team members as was the case in this study.

## Conclusions

Although studies vary in their definition of spirituality, the spiritual dimension in parents of children with life-limiting or life-threatening conditions becomes manifest in how parents connect to their faith, others, and themselves as parents. Healthcare professionals can support parents by being sensitive to the spiritual process parents are going through. The way in which healthcare professionals can provide spiritual care to the parents of a seriously ill child requires more research in the natural setting of clinical practice. Quantitative and qualitative research methods are needed to explore further the spiritual process of parents, how professionals can respond to this spiritual process in daily practice, in which situations expertise from healthcare professionals specialized in palliative care or spiritual and grief counselors is needed and in what way spiritual care in pediatric palliative care can best be organized in diverse care settings.

## Supplemental Material

sj-docx-1-pmj-10.1177_02692163231186173 – Supplemental material for The spiritual dimension of parenting a child with a life-limiting or life-threatening condition: A mixed-methods systematic review Click here for additional data file. Supplemental material, sj-docx-1-pmj-10.1177_02692163231186173 for The spiritual dimension of parenting a child with a life-limiting or life-threatening condition: A mixed-methods systematic review by Marijanne Engel, Marije A Brouwer, Nienke Jansen, Carlo Leget, Saskia CCM Teunissen and Marijke C Kars in Palliative Medicine
