# Supplementary material for: The spiritual dimension of parenting a child with a life-limiting or life-threatening condition: A mixed-methods systematic review
Source: Palliat Med. 2023 Jul 17;37(9):1303–25. doi: 10.1177/02692163231186173 (PMC10548770; doi:10.1177/02692163231186173)
Supplement: sj-docx-1-pmj-10.1177_02692163231186173 – Supplemental material for The spiritual dimension of parenting a child with a life-limiting or life-threatening condition: A mixed-methods systematic review [file sj-docx-1-pmj-10.1177_02692163231186173.docx]

**The spiritual dimension of parenting a child with a life-limiting or life-threatening condition: a mixed-methods systematic review**

**Supplemental file 1 Database search strategy**

PubMed

("palliative care"[MeSH Terms] OR "terminal care"[MeSH Terms] OR "hospice care"[MeSH Terms] OR "palliative medicine"[MeSH Terms] OR "life support care"[MeSH Terms] OR "hospice and palliative care nursing"[MeSH Terms] OR "life change events"[MeSH Terms] OR "advance care planning"[MeSH Terms] OR "palliati*"[Title/Abstract] OR "end of life"[Title/Abstract] OR "Terminal"[Title/Abstract] OR "terminally ill"[Title/Abstract] OR "end stage disease*"[Title/Abstract] OR "Hospice"[Title/Abstract] OR "Death"[Title/Abstract] OR "life threatening"[Title/Abstract] OR "life limit*"[Title/Abstract] OR "bereave*"[Title/Abstract] OR "Dying"[Title/Abstract] OR "severe ill*"[Title/Abstract]) AND ("child"[MeSH Terms] OR "infant"[MeSH Terms] OR "pediatrics"[MeSH Terms] OR "child*"[Title/Abstract] OR "infan*"[Title/Abstract] OR "newborn*"[Title/Abstract] OR "new born*"[Title/Abstract] OR "perinat*"[Title/Abstract] OR "neonat*"[Title/Abstract] OR "baby*"[Title/Abstract] OR "Babies"[Title/Abstract] OR "toddler*"[Title/Abstract] OR "schoolchild*"[Title/Abstract] OR "school child*"[Title/Abstract] OR "adolescen*"[Title/Abstract] OR "pediatric*"[Title/Abstract] OR "peadiatr*"[Title/Abstract] OR "Boy"[Title/Abstract] OR "Boys"[Title/Abstract] OR "Girl"[Title/Abstract] OR "Girls"[Title/Abstract] OR "Youth"[Title/Abstract] OR "Youths"[Title/Abstract] OR "Teen"[Title/Abstract] OR "Teens"[Title/Abstract] OR "teenager*"[Title/Abstract] OR "Puberty"[Title/Abstract] OR "minors"[Title/Abstract] OR "Kid"[Title/Abstract] OR "Kids"[Title/Abstract] OR "under age*"[Title/Abstract]) AND ("parents"[MeSH Terms] OR "family"[MeSH Terms] OR "caregivers"[MeSH Terms] OR "parent*"[Title/Abstract] OR "mother*"[Title/Abstract] OR "father*"[Title/Abstract] OR "famil*"[Title/Abstract] OR "matern*"[Title/Abstract] OR "patern*"[Title/Abstract] OR "stepmother*"[Title/Abstract] OR "stepfather*"[Title/Abstract] OR "caregiver*"[Title/Abstract]) AND ("religion and psychology"[MeSH Terms] OR "spiritual therapies"[MeSH Terms] OR "holistic health"[MeSH Terms] OR "existentialism"[MeSH Terms] OR "existentia*"[Title/Abstract] OR "meaning*"[Title/Abstract] OR "spiritual*"[Title/Abstract] OR "religio*"[Title/Abstract] OR "Faith"[Title/Abstract] OR "Pastoral"[Title/Abstract] OR "mindful*"[Title/Abstract] OR "pray*"[Title/Abstract] OR "Social"[Title/Abstract] OR “Holistic”[Title/Abstract]) AND ("Need*"[Title/Abstract] OR "issue"[Title/Abstract] OR "experience"[Title/Abstract] OR "dilemma"[Title/Abstract] OR "wish"[Title/Abstract] OR "demand"[Title/Abstract] OR "burden"[Title/Abstract] OR "preference*"[Title/Abstract] OR "unmet"[Title/Abstract] OR "help*"[Title/Abstract])

Cochrane

(([mh "palliative care"] OR [mh "terminal care"] OR [mh "hospice care"] OR [mh "palliative medicine"] OR [mh "life support care"] OR [mh "hospice and palliative care nursing"] OR [mh "life change events"] OR [mh "advance care planning"] OR palliati*:ti,ab,kw OR "end of life":ti,ab,kw OR Terminal:ti,ab,kw OR "terminally ill":ti,ab,kw OR ("end stage" NEXT disease*):ti,ab,kw OR Hospice:ti,ab,kw OR Death:ti,ab,kw OR "life threatening":ti,ab,kw OR ("life" NEXT limit*):ti,ab,kw OR bereave*:ti,ab,kw OR Dying:ti,ab,kw OR ("severe" NEXT ill*):ti,ab,kw) AND ([mh child] OR [mh infant] OR [mh pediatrics] OR child*:ti,ab,kw OR infan*:ti,ab,kw OR newborn*:ti,ab,kw OR ("new" NEXT born*):ti,ab,kw OR perinat*:ti,ab,kw OR neonat*:ti,ab,kw OR baby*:ti,ab,kw OR Babies:ti,ab,kw OR toddler*:ti,ab,kw OR schoolchild*:ti,ab,kw OR ("school" NEXT child*):ti,ab,kw OR adolescen*:ti,ab,kw OR pediatric*:ti,ab,kw OR peadiatr*:ti,ab,kw OR Boy:ti,ab,kw OR Boys:ti,ab,kw OR Girl:ti,ab,kw OR Girls:ti,ab,kw OR Youth:ti,ab,kw OR Youths:ti,ab,kw OR Teen:ti,ab,kw OR Teens:ti,ab,kw OR teenager*:ti,ab,kw OR Puberty:ti,ab,kw OR minors:ti,ab,kw OR Kid:ti,ab,kw OR Kids:ti,ab,kw OR ("under" NEXT age*):ti,ab,kw) AND ([mh parents] OR [mh family] OR [mh caregivers] OR parent*:ti,ab,kw OR mother*:ti,ab,kw OR father*:ti,ab,kw OR famil*:ti,ab,kw OR matern*:ti,ab,kw OR patern*:ti,ab,kw OR ("step" NEXT mother*):ti,ab,kw OR ("step" NEXT father*):ti,ab,kw OR caregiver*:ti,ab,kw) AND ([mh "religion and psychology"] OR [mh "spiritual therapies"] OR [mh "holistic health"] OR [mh existentialism] OR existentia*:ti,ab,kw OR meaning*:ti,ab,kw OR spiritual*:ti,ab,kw OR religio*:ti,ab,kw OR Faith:ti,ab,kw OR Pastoral:ti,ab,kw OR mindful*:ti,ab,kw OR pray*:ti,ab,kw OR Social:ti,ab,kw OR Holistic:ti,ab,kw) AND (Need*:ti,ab,kw OR issue:ti,ab,kw OR experience:ti,ab,kw OR dilemma:ti,ab,kw OR wish:ti,ab,kw OR demand:ti,ab,kw OR burden:ti,ab,kw OR preference*:ti,ab,kw OR unmet:ti,ab,kw OR help*:ti,ab,kw))

CINAHL

((MH "palliative care"+) OR (MH "terminal care"+) OR (MH "hospice care"+) OR (MH "palliative medicine"+) OR (MH "life support care"+) OR (MH "hospice and palliative care nursing"+) OR (MH "life change events"+) OR (MH "advance care planning"+) OR (TI palliati* OR AB palliati*) OR (TI "end of life" OR AB "end of life") OR (TI Terminal OR AB Terminal) OR (TI "terminally ill" OR AB "terminally ill") OR (TI "end stage disease*" OR AB "end stage disease*") OR (TI Hospice OR AB Hospice) OR (TI Death OR AB Death) OR (TI "life threatening" OR AB "life threatening") OR (TI "life limit*" OR AB "life limit*") OR (TI bereave* OR AB bereave*) OR (TI Dying OR AB Dying) OR (TI "severe ill*" OR AB "severe ill*")) AND ((MH child+) OR (MH infant+) OR (MH pediatrics+) OR (TI child* OR AB child*) OR (TI infan* OR AB infan*) OR (TI newborn* OR AB newborn*) OR (TI "new born*" OR AB "new born*") OR (TI perinat* OR AB perinat*) OR (TI neonat* OR AB neonat*) OR (TI baby* OR AB baby*) OR (TI Babies OR AB Babies) OR (TI toddler* OR AB toddler*) OR (TI schoolchild* OR AB schoolchild*) OR (TI "school child*" OR AB "school child*") OR (TI adolescen* OR AB adolescen*) OR (TI pediatric* OR AB pediatric*) OR (TI peadiatr* OR AB peadiatr*) OR (TI Boy OR AB Boy) OR (TI Boys OR AB Boys) OR (TI Girl OR AB Girl) OR (TI Girls OR AB Girls) OR (TI Youth OR AB Youth) OR (TI Youths OR AB Youths) OR (TI Teen OR AB Teen) OR (TI Teens OR AB Teens) OR (TI teenager* OR AB teenager*) OR (TI Puberty OR AB Puberty) OR (TI minors OR AB minors) OR (TI Kid OR AB Kid) OR (TI Kids OR AB Kids) OR (TI "under age*" OR AB "under age*")) AND ((MH parents+) OR (MH family+) OR (MH caregivers+) OR (TI parent* OR AB parent*) OR (TI mother* OR AB mother*) OR (TI father* OR AB father*) OR (TI famil* OR AB famil*) OR (TI matern* OR AB matern*) OR (TI patern* OR AB patern*) OR (TI "stepmother*" OR AB "stepmother*") OR (TI "stepfather*" OR AB "stepfather*") OR (TI caregiver* OR AB caregiver*)) AND ((MH "religion and psychology"+) OR (MH "spiritual therapies"+) OR (MH "holistic health"+) OR (MH existentialism+) OR (TI existentia* OR AB existentia*) OR (TI meaning* OR AB meaning*) OR (TI spiritual* OR AB spiritual*) OR (TI religio* OR AB religio*) OR (TI Faith OR AB Faith) OR (TI Pastoral OR AB Pastoral) OR (TI mindful* OR AB mindful*) OR (TI pray* OR AB pray*) OR (TI Social OR AB Social) OR (TI Holistic OR AB Holistic)) AND ((TI Need* OR AB Need*) OR (TI issue OR AB issue) OR (TI experience OR AB experience) OR (TI dilemma OR AB dilemma) OR (TI wish OR AB wish) OR (TI demand OR AB demand) OR (TI burden OR AB burden) OR (TI preference* OR AB preference*) OR (TI unmet OR AB unmet) OR (TI help* OR AB help*))

PsycInfo

#1

exp palliative care/ OR exp terminally ill patients/ OR exp hospice/ OR exp life sustaining treatment/ OR exp life changes/ OR exp treatment planning/ OR palliati*.ti,ab,id. OR "end of life".ti,ab,id. OR terminal.ti,ab,id. OR "terminally ill".ti,ab,id. OR "end stage disease*".ti,ab,id. OR hospice.ti,ab,id. OR death.ti,ab,id. OR "life threatening".ti,ab,id. OR "life threatening".ti,ab,id. OR "life limit*".ti,ab,id. OR bereave*.ti,ab,id. OR dying.ti,ab,id. OR "severe ill*".ti,ab,id.

#2

exp child care/ OR exp child care workers/ OR exp child death/ OR exp child health/ OR exp parent child communication/OR exp parent child relations/ OR exp pediatrics/ OR child*.ti,ab,id. OR infan*.ti,ab,id. OR newborn*.ti,ab,id. OR "new born*".ti,ab,id. OR perinat*.ti,ab,id. OR neonat*.ti,ab,id. OR baby*.ti,ab,id. OR babies.ti,ab,id. OR toddler*.ti,ab,id. OR schoolchild*.ti,ab,id. OR "school child*".ti,ab,id. OR adolescen*.ti,ab,id. OR pediatric*.ti,ab,id. OR peadiatr*.ti,ab,id. OR boy.ti,ab,id. OR boys.ti,ab,id. OR girl.ti,ab,id. OR girls.ti,ab,id. OR youth.ti,ab,id. OR youths.ti,ab,id. OR teen.ti,ab,id. OR teens.ti,ab,id. OR teenager*.ti,ab,id. OR puberty.ti,ab,id. OR minors.ti,ab,id. OR kid.ti,ab,id. OR kids.ti,ab,id. OR "under age*".ti,ab,id.

#3

exp parents/ OR exp family/ OR exp caregivers/ OR parent*.ti,ab,id. OR mother*.ti,ab,id. OR father*.ti,ab,id. OR famil*.ti,ab,id. OR matern*.ti,ab,id. OR patern*.ti,ab,id. OR "stepmother*".ti,ab,id. OR "stepfather*".ti,ab,id. OR caregiver*.ti,ab,id.

#4

exp spirituality/ OR exp holistic health/ OR exp existentialism/ OR existentia*.ti,ab,id. OR meaning*.ti,ab,id. OR spiritual*.ti,ab,id. OR religio*.ti,ab,id. OR faith.ti,ab,id. OR pastoral.ti,ab,id. OR mindful*.ti,ab,id. OR pray*.ti,ab,id. OR social.ti,ab,id. OR holistic.ti,ab,id.

#5

need*.ti,ab,id. OR issue.ti,ab,id. OR experience.ti,ab,id. OR dilemma.ti,ab,id. OR wish.ti,ab,id. OR demand.ti,ab,id. OR burden.ti,ab,id. OR preference*.ti,ab,id. OR unmet.ti,ab,id. OR help*.ti,ab,id.

1 AND 2 AND 3 AND 4 AND 5

Embase

(‘palliative care’/exp OR ‘terminally ill patients’/exp OR hospice/exp OR ‘life sustaining treatment’/exp OR ‘life changes’/exp OR ‘treatment planning’/exp OR palliati*:ti,ab,kw OR ‘end of life’:ti,ab,kw OR terminal:ti,ab,kw OR ‘terminally ill’:ti,ab,kw OR ‘end stage disease*’:ti,ab,kw OR hospice:ti,ab,kw OR death:ti,ab,kw OR ‘life threatening’:ti,ab,kw OR ‘life threatening’:ti,ab,kw OR ‘life limit*’:ti,ab,kw OR bereave*:ti,ab,kw OR dying:ti,ab,kw OR ‘severe ill*’:ti,ab,kw) AND (‘child care’/exp OR ‘child care workers’/exp OR ‘child death’/exp OR ‘child health’/exp OR ‘parent child communication’/exp OR ‘parent child relations’/exp OR pediatrics/exp OR child*:ti,ab,kw OR infan*:ti,ab,kw OR newborn*:ti,ab,kw OR ‘new born*’:ti,ab,kw OR perinat*:ti,ab,kw OR neonat*:ti,ab,kw OR baby*:ti,ab,kw OR babies:ti,ab,kw OR toddler*:ti,ab,kw OR schoolchild*:ti,ab,kw OR ‘school child*’:ti,ab,kw OR adolescen*:ti,ab,kw OR pediatric*:ti,ab,kw OR peadiatr*:ti,ab,kw OR boy:ti,ab,kw OR boys:ti,ab,kw OR girl:ti,ab,kw OR girls:ti,ab,kw OR youth:ti,ab,kw OR youths:ti,ab,kw OR teen:ti,ab,kw OR teens:ti,ab,kw OR teenager*:ti,ab,kw OR puberty:ti,ab,kw OR minors:ti,ab,kw OR kid:ti,ab,kw OR kids:ti,ab,kw OR ‘under age*’:ti,ab,kw) AND (parents/exp OR family/exp OR caregivers/exp OR parent*:ti,ab,kw OR mother*:ti,ab,kw OR father*:ti,ab,kw OR famil*:ti,ab,kw OR matern*:ti,ab,kw OR patern*:ti,ab,kw OR ‘stepmother*’:ti,ab,kw OR ‘stepfather*’:ti,ab,kw OR caregiver*:ti,ab,kw) AND (spirituality/exp OR ‘holistic health’/exp OR existentialism/exp OR existentia*:ti,ab,kw OR meaning*:ti,ab,kw OR spiritual*:ti,ab,kw OR religio*:ti,ab,kw OR faith:ti,ab,kw OR pastoral:ti,ab,kw OR mindful*:ti,ab,kw OR pray*.ti,ab,kw. OR social:ti,ab,kw OR holistic:ti,ab,kw) AND (need*:ti,ab,kw OR issue:ti,ab,kw OR experience:ti,ab,kw OR dilemma:ti,ab,kw OR wish:ti,ab,kw OR demand:ti,ab,kw OR burden:ti,ab,kw OR preference*:ti,ab,kw OR unmet:ti,ab,kw OR help*:ti,ab,kw)

**Supplemental file 2 Quality assessment studies**

**Table A.2.1 Quality assessment qualitative studies with CASP**

|  | **Aim** | **Methodology** | **Design** | **Recruitment** | **Data collection** | **Relationship** | **Ethical** | **Data analysis** | **Finding** | **Values** | **Score** |
| --- | --- | --- | --- | --- | --- | --- | --- | --- | --- | --- | --- |
| Akaberian et al. (2021) | Yes | Yes | Yes | Yes | Yes | No | Yes | Yes | Yes | Valuable | 9 |
| Arora et al. (2021) | Yes | Yes | Can’t tell | Yes | Yes | Yes | Yes | Yes | Yes | Valuable | 9.5 |
| Atashzadeh-Shoorideh et al. (2018) | Yes | Yes | Yes | Can’t tell | Yes | No | Can’t tell | Yes | Yes | Valuable | 8 |
| Bally et al. (2021) | Yes | Yes | Yes | Can’t tell | Yes | Yes | Yes | Yes | Yes | Valuable | 9.5 |
| Bogetz et al. (2021) | Yes | Yes | Yes | Can’t tell | Yes | No | Yes | Yes | Yes | Valuable | 8.5 |
| Cai, S., et al. (2020) | Yes | Yes | Yes | Yes | Yes | No | Yes | Yes | Yes | Valuable | 9 |
| Cho-Hee et al. (2022) | Yes | Yes | Yes | Yes | Yes | No | Yes | Can’t tell | Yes | Valuable | 8.5 |
| Chong L. et al. (2022) | Yes | Yes | Can’t tell | Can’t tell | Yes | Yes | Yes | Yes | Yes | Valuable | 9 |
| Chong P.H. et al. (2021) | Yes | Yes | Yes | Yes | Yes | No | Yes | Yes | Yes | Valuable | 9 |
| Coad et al. (2015) | Yes | Yes | Yes | Can’t tell | Can’t tell | No | Yes | Yes | Yes | Valuable | 8 |
| Collins et al. (2016) | Yes | Yes | Yes | Yes | Yes | Yes | Yes | Yes | Yes | Valuable | 10 |
| Courtney et al. (2018) | Yes | Yes | Can’t tell | Yes | Can’t tell | No | Yes | Can’t tell | Yes | Valuable | 7.5 |
| Dantas Jesuíno da Costa et al. (2018) | Yes | Yes | Yes | Yes | Can’t tell | No | Yes | Yes | Yes | Valuable | 8.5 |
| De Clercq et al. (2017) | Yes | Yes | Yes | Can’t tell | Can’t tell | No | Yes | Yes | Yes | Valuable | 8 |
| Donovan et al. (2022) | Yes | Yes | Yes | Yes | Yes | Yes | Yes | Yes | Yes | Valuable | 10 |
| Dos Santos et al. (2020) | Yes | Yes | Yes | Yes | Yes | No | Yes | Yes | Yes | Valuable | 9 |
| Doumit et al. (2019) | Yes | Yes | Yes | Yes | Yes | No | Can’t tell | Yes | Yes | Valuable | 8.5 |
| Effendy et al. (2022) | Yes | Yes | Yes | Can’t tell | Yes | No | Yes | Yes | Can’t tell | Valuable | 8 |
| Falkenburg et al.  (2020) | Yes | Yes | Can’t tell | Yes | Yes | Yes | Can’t tell | Yes | Yes | Valuable | 9 |
| Freitas et al. (2017) | Yes | Yes | Can’t tell | Can’t tell | Yes | No | Yes | Yes | Yes | Valuable | 8 |
| Hafez et al. (2021) | Yes | Yes | Can’t tell | Yes | Yes | No | Yes | Yes | Yes | Valuable | 8.5 |
| Higgs et al. (2016) | Yes | Yes | Can’t tell | Yes | Yes | Yes | Yes | Yes | Yes | Valuable | 9.5 |
| Hurley et al. (2021) | Yes | Yes | Yes | Yes | Yes | No | Yes | Yes | Yes | Valuable | 9 |
| Jordan et al. (2015) | Yes | Yes | Yes | Yes | Yes | No | Can’t tell | Yes | Yes | Valuable | 8.5 |
| Jyothi Cornelio et al. (2016) | Yes | Can’t tell | Yes | No | Yes | No | Yes | Yes | Yes | Valuable | 7.5 |
| Kamihara et al.  (2015) | Yes | Can’t tell | No | Yes | Yes | No | Yes | Yes | Yes | Valuable | 7.5 |
| Koch et al. (2022) | Yes | Yes | Can’t tell | Yes | Yes | No | Can’t tell | Yes | Yes | Valuable | 8 |
| Lin et al. (2020) | Yes | Yes | Can’t tell | Yes | Yes | Yes | Yes | Yes | Yes | Valuable | 9.5 |
| Lotz et al. (2017) | Yes | Yes | Can’t tell | Yes | Yes | Yes | Yes | Yes | Yes | Valuable | 9.5 |
| Lou et al. (2015) | Yes | Yes | Yes | No | Yes | No | Yes | Yes | Yes | Valuable | 8 |
| Love et al. (2022) | Yes | Yes | Can’t tell | Yes | Yes | Yes | Yes | Yes | Yes | Valuable | 9.5 |
| Malcolm et al. (2021) | Yes | Yes | Yes | Yes | Yes | Yes | Yes | Yes | Yes | Valuable | 10 |
| Misko et al. (2015) | Yes | Yes | Yes | No | Yes | No | Yes | Can’t tell | Yes | Valuable | 7.5 |
| Nafratilova et al. (2018) | Yes | No | Can’t tell | Can’t tell | Can’t tell | No | Yes | Can’t tell | Can’t tell | Valuable | 5.5 |
| Nicholas et al. (2017) | Yes | Yes | Yes | Yes | Yes | No | Yes | Yes | Yes | Valuable | 9 |
| Palacios-Espinosa et al. (2021) | Yes | Yes | Yes | Can’t tell | Yes | No | Yes | Yes | Yes | Valuable | 8.5 |
| Pishkuhi et al. (2018) | Yes | Yes | Yes | Yes | Yes | No | No | Yes | Yes | Valuable | 8 |
| Schaefer et al.  (2021) | Yes | Yes | No | Can’t tell | Yes | Yes | Can’t tell | Yes | Yes | Valuable | 8 |
| Silva et al. (2019) | Yes | Can’t tell | Yes | No | Yes | No | Yes | Yes | Yes | Valuable | 7.5 |
| Smith et al. (2018) | Yes | Yes | Yes | Can’t tell | Yes | No | Yes | Yes | Yes | Valuable | 8.5 |
| Somanadhan and Larkin (2016) | Yes | Yes | Yes | Yes | Yes | No | Yes | Yes | Yes | Valuable | 9 |
| Szabat (2020) | Yes | Yes | No | Yes | Yes | No | Yes | Yes | Yes | Valuable | 8 |
| Taib et al.(2021) | Yes | Yes | Yes | Can’t tell | Yes | Yes | Yes | Can’t tell | Yes | Valuable | 9 |
| Verberne et al. (2019) | Yes | Yes | Yes | Yes | Yes | Yes | Yes | Yes | Yes | Valuable | 10 |
| Wang et al. (2019) | Yes | Yes | Yes | Yes | Yes | No | Yes | Yes | Yes | Valuable | 9 |
| Weaver et al. (2021) | Yes | No | Yes | No | No | No | No | Can’t tell | Yes | Valuable | 4.5 |
| Yang et al. (2016) | Yes | Yes | Yes | Can’t tell | Yes | No | Yes | Yes | Yes | Valuable | 8.5 |

**Table A.2.2 Cochrane adapted Risk of bias assessment observational studies**

|  | **Selection process of study population** | **Comparability of compared groups (controlled studies or in time)** | **Standardized protocol for the determinant** | **Standardized protocol for measuring the outcome** | **Missing data with regard to inclusion or follow up/**  **incomplete outcome data** | **Adjustment for confounders** | **Selective outcome reporting** | **Total score** |
| --- | --- | --- | --- | --- | --- | --- | --- | --- |
| **Arutyunyan et al.**  **(2018)** | + | NA | NA | + | - | NA | + | 3 |
| **Bedoya et al. (2021)** | + | NA | NA | - | ? | NA | ? | 1 |
| **Boyden et al. (2021)** | + | NA | - | + | - | NA | + | 3 |
| **Czyżowska et al. (2021)** | + | NA | NA | + | - - | NA | + | 3 |
| **Janvier et al. (2016)** | - | NA | NA | + | - - | - | + | 2 |
| **Kelly et al. (2016)** | - - | NA | NA | + | - | - | + | 2 |
| **Latha et al. (2016)** | - | NA | NA | + |  | - | + | 2 |
| **Picci et al. (2015)** | + | + | NA | + | ? | NA | + | 4 |
| **Rao et al. (2022)** | ? | NA | NA | + | - | NA | ? | 1 |
| **Siden and Steele (2015)** | + | NA | NA | + | + | - | + | 4 |
| **Wiener et al. (2020)** | + | NA | NA | + | + | NA | - | 3 |

NA = not applicable; + = criterion with low risk of bias, a score of 1 was assigned; - criterion with high risk of bias, a score of 0 was assigned; ? = criterion with unclear risk of bias, no score was assigned

**Table A.2.3 Mixed methods studies, adapted risk of bias assessment quantitative part**

|  | **Selection process of study population** | **Comparability of compared groups**  **(controlled studies or in time)** | **Standardized protocol for determinant ( )** | **Standardized protocol for**  **measuring the outcome** | **Missing data with regard to inclusion or**  **follow up/ incomplete outcome data** | **Adjustment for confounders** | **Selective outcome reporting** | **Total score** |
| --- | --- | --- | --- | --- | --- | --- | --- | --- |
| **Eskola et al. (2017)** | + | - | - | + | - | NA | - | 2 |
| **Haley et al. (2016)** | - | NA | + | + | ? | NA | - | 2 |
| **Michelson et al. (2022)** | + | NA | ? | NA | + | NA | + | 3 |
| **Mooney-Doyle et al. (2018)** | + | NA | NA | + | + | NA | - | 3 |
| **Zimmermann et al. (2022)** | + | NA | NA | ? | NA | + | + | 3 |

NA = not applicable; + = criterion with low risk of bias, a score of 1 was assigned; - criterion with high risk of bias, a score of 0 was assigned; ? = criterion with unclear risk of bias,

no score was assigned

**Table A.2.4 Mixed methods studies, quality assessment qualitative part, CASP**

|  | **Aim** | **Methodology** | **Design** | **Recruitment** | **Data collection** | **Relationship** | **Ethical** | **Data analysis** | **Finding** | **Values** | **Score** |
| --- | --- | --- | --- | --- | --- | --- | --- | --- | --- | --- | --- |
| **Eskola et al. (2017)** | Yes | Yes | Yes | Yes | Yes | No | Yes | Yes | Yes | Valuable | 9 |
| **Haley et al. (2016)** | Yes | Can’t tell | Yes | No | Yes | No | Can’t tell | No | Yes | Can’t tell | 5.5 |
| **Michelson et al. (2022)** | Yes | Yes | Can’t tell | Yes | Yes | No | Yes | Can’t tell | Can’t tell | Valuable | 7,5 |
| **Mooney-Doyle et al. (2018)** | Yes | Yes | Yes | Yes | Yes | No | Yes | Yes | Yes | Valuable | 9 |
| **Zimmermann et al.**  **(2022)** | Yes | Yes | Yes | Yes | Yes | Yes | Yes | Yes | Yes | Valuable | 10 |

**Supplemental file 3 Description of spirituality in included articles**

| **Author (year)** | **Dimensions of spirituality from the EAPC definition identified in the definition or description of spirituality in included studies** | | | |  |
| --- | --- | --- | --- | --- | --- |
|  | Existential questions | Value based considerations and attitudes | | Religious considerations and foundations |  |
| Akaberian et al. (2021); |  | X | | X |  |
| Arutyunyan et al. (2018) |  | X | | X |  |
| Atashzadeh-Shoorideh et al. (2018) | X | X | | X |  |
| Cai et al. (2020) | X | X | | X |  |
| Czyżowska, N., et al. (2021) | X | X | | X |  |
| Doumit et al. (2019) | X | X | | X |  |
| Falkenburg et al. (2020) | X | X | | X |  |
| Freitas et al. (2017) | X | X | | X |  |
| Hafez et al. (2021) |  |  | | X |  |
| Lin et al. (2020) |  | X | | X |  |
| Nicholas et al. (2017) | X | X | | X |  |
| Malcolm et al. (2021) |  |  | | X |  |
| Schaefer et al. (2021) |  |  | | X |  |
| Szabat (2020) |  |  | | X |  |
| **No conceptual definition or description of spirituality provided** | | | | | **Number of studies**  **(n total)** |
| Arora et al. (2021); Bally et al. (2021); Bogetz et al. (2021); Cho-Hee et al. (2022); Chong L. et al. (2022); Chong P.H. et al. (2021); Courtney et al. (2018); De Clercq et al. (2017); Donovan et al. (2022); Effendy et al. (2022); Eskola et al. (2017); Haley et al. (2016); Kelly et al. (2016); Koch et al. (2022); Lotz et al. (2017); Lou et al. (2015); Michelson et al. (2022); Misko et al. (2015); Mooney-Doyle et al. (2018); Nafratilova et al. (2018); Palacios-Espinosa et al. (2021); Pishkuhi et al. (2018); Rao et al. (2022) Silva Eder Dias et al. (2019); Siden and Steele (2015); Smith et al. (2018); Taib et al. (2021); Yang et al. (2016); Zimmermann et al. (2022) | | | The concept ‘spirituality’ is explicitly mentioned, but not specified. Themes related to spirituality are described. | | 29 |
| Bedoya et al. (2021); Boyden et al. (2021); Coad et al. (2016); Collins et al. (2016); Dantas Jesuíno da Costa et al. (2018); Dos Santos et al. (2020); Higgs et al. (2016); Hurley et al. (2021); Janvier et al. (2016); Jordan et al. (2015); Jyothi Cornelio et al. (2016); Kamihara et al. (2015); Latha et al. (2016); Love et al. (2022); Picci et al. (2015); Somanadhan and Larkin (2016); Verberne et al. (2019); Wang et al. (2019); Weaver et al. (2021); Wiener et al. (2020) | | | The concept ‘spirituality’ is not mentioned, but themes related to spirituality are described. | | 20 |
